# Replication of Extended Lifespan Phenotype in Mice with Deletion of Insulin Receptor Substrate 1

**DOI:** 10.1371/journal.pone.0016144

**Published:** 2011-01-25

**Authors:** Colin Selman, Linda Partridge, Dominic J. Withers

**Affiliations:** 1 Institute of Biological and Environmental Sciences, School of Biological Sciences, University of Aberdeen, Aberdeen, United Kingdom; 2 Institute of Healthy Ageing, Department of Genetics, Evolution and Environment, University College London, London, United Kingdom; 3 Metabolic Signaling Group, Medical Research Council Clinical Sciences Centre, Imperial College, London, United Kingdom; University of Washington, United States of America

## Abstract

We previously reported that global deletion of insulin receptor substrate protein 1 (*Irs1*) extends lifespan and increases resistance to several age-related pathologies in female mice. However, no effect on lifespan was observed in male *Irs1* null mice. We suggested at the time that the lack of any effect in males might have been due to a sample size issue. While such lifespan studies are essential to our understanding of the aging process, they are generally based on survival curves derived from single experiments, primarily due to time and economic constraints. Consequently, the robustness of such findings as a basis for further investigation has been questioned. We have therefore measured lifespan in a second, separate cohort of *Irs1* null female mice, and show that, consistent with our previous finding, global deletion of *Irs1* significantly extends lifespan in female mice. In addition, an augmented and completed study demonstrates lifespan extension in male *Irs1* null mice. Therefore, we show that reduced IRS1-dependent signalling is a robust mechanism through which mammalian lifespan can be modulated.

## Introduction

The precise mechanisms underlying the aging process in multicellular organisms are currently unknown. However, the insulin/insulin-like growth factor-1 (IGF-1) signalling (IIS) pathway and mammalian target of rapamycin (mTOR) signalling pathway are highly conserved candidates for protection against the effects of the ageing process [1], [2], [3], [4], [5], [6]. Reduced IIS extends lifespan in both invertebrate and vertebrate model organisms [2], [3], [7]. In addition, extended lifespan in growth hormone (GH)/GH-receptor deficient dwarf mice may act through IIS attenuation [8], and polymorphisms in several IIS and GH-related genes have been shown to correlate with human lifespan [9], [10], [11].

Recently, we reported that global deletion of insulin receptor substrate protein 1 (*Irs1*), a key downstream mediator of IIS, increased lifespan in female mice relative to wild type (WT) controls [12]. In addition, we demonstrated that this long life was accompanied by a resistance to several age-related pathologies including skin, bone and motor dysfunction and protection against age-related glucose intolerance [12]. However, no difference was reported in male *Irs1* null (*Irs1^−/−^*) mice compared to wild-type (*WT*) controls. In our earlier paper [12] we suggested that this lack of a lifespan effect in males may be partly explained by the relatively small number of male *Irs1* ^−/−^ mice used (n = 10), and/or by the fact that 3 individuals from this group were still alive at the time of publication. Interestingly, despite our previously reported lack of any lifespan effect in male *Irs1* ^−/−^ mice, these animals, like females *Irs1* ^−/−^ mice, were protected against late life pathologies including complete protection against the ulcerative dermatitis seen in aged male WT controls [12]. Male *Irs1* ^−/−^ mice also were protected against an age-associated alteration in T cell populations (increase in memory T cells, decrease in naive T cells) [12].

As the majority of IIS mutant mouse aging studies are based on lifespan curves derived from single experimental cohorts, several authors (e.g. [13], [14]) have suggested that replication and validation of these experiments is necessary in order to use these models with confidence in our quest to understand the aging process. Indeed, significant effort and resources may be wasted if any model subsequently shows no repeatable retardation in aging. Ladiges *et al.* [14] recently reported that only 3 (Ames (*Prop1^df/df^*), Snell (*Pit1^dw/dw^*), growth hormone receptor knockout (*GHR-KO*)) out of 20 published long-lived mouse models showed consistent lifespan extension across separate studies. In pure IIS mutants the situation appears more ambiguous, as mice heterozygous for a null allele of insulin receptor substrate protein 2 (*Irs2*) were reported as being long-lived [15], but this finding was not replicated in a second study [16]. The reasons for this discrepancy between these two studies are currently unclear [16], but may be due to an atypical WT survival curve in the earlier study [15].

In the current study we examined lifespan in a second, independent cohort of female WT and *Irs1^−/−^* mice to determine whether our original finding was indeed repeatable. In addition, we present data from our completed male lifespan study which has been further augmented by 2 additional WT and 2 additional *Irs1^−/−^* littermates that were not included in the original study, but were derived from the same group of breeders as that of our original study.

## Results and Discussion

**Figure 1:**
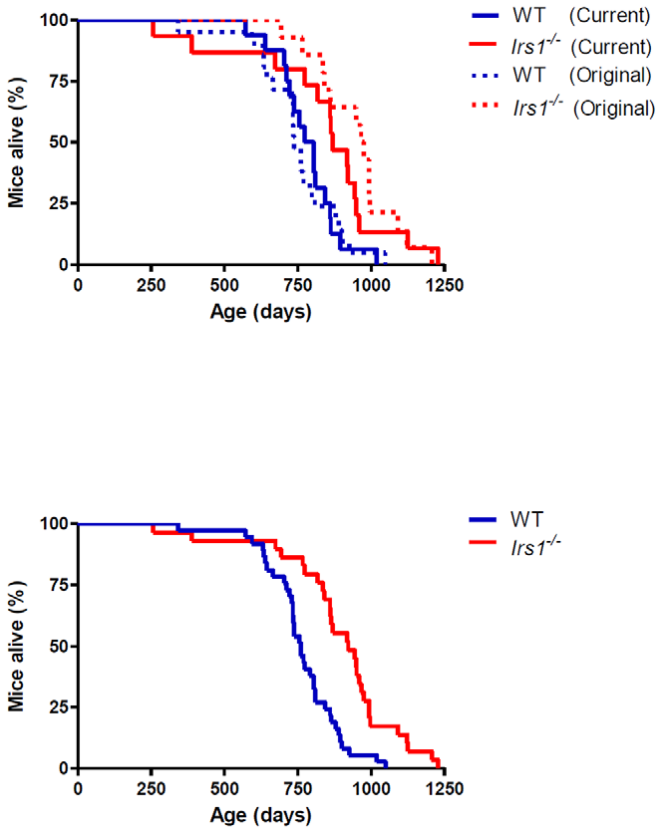
Female *Irs1^−/−^* mice are significantly long-lived. Kaplan-Meier survival curves for female WT and *Irs1^−/−^* mice from this current study (Solid lines; under husbandry conditions exactly as previously described [12], [16]) and our original study (Stippled lines; [12]). Blue and red lines indicate WT and *Irs1^−/−^* mice respectively (**A**). Kaplan-Meier survival curves for the combined data for female WT and *Irs1^−/−^* mice from this current study and our original study ([12]). Blue and red lines indicate WT and *Irs1^−/−^* mice respectively (**B**).

**Figure 2:**
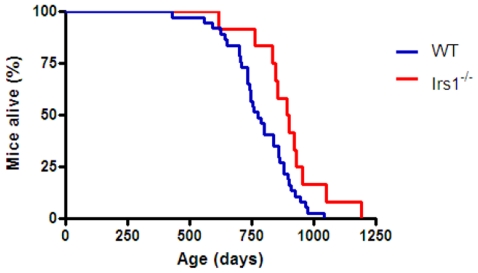
Male *Irs1^−/−^* mice are significantly long-lived. Kaplan-Meier survival curves for male WT and *Irs1^−/−^* mice. Blue and red lines indicate WT and *Irs1^−/−^* mice respectively.

In agreement with our previous study [12], median lifespan was significantly increased (Log rank X^2^ = 4.916, P<0.05) in female *Irs1^−/−^* mice relative to WT controls (Fig. 1A (indicated by solid lines), Table 1). No difference in lifespan was observed within each genotype (i.e. WT vs. WT; *Irs1^−/−^* vs. *Irs1^−/−^*) between the current study and our original study [12] (Fig. 1A; WT, X^2^ = 0.020, P>0.05; *Irs1^−/−^*, X^2^ = 0.805, P>0.05). Cox regression analysis on combined data from both independent female studies (current vs. original study [12]) indicated that date of birth (Z = 1.522, P>0.05), parental identity (Z = 0.313, P>0.05) or experimental cohort (Z = 1.233, P>0.05) did not have any significant impact on lifespan. However, the effect of genotype on lifespan was highly significant (Z = 14.557, P<0.001). When the mortality data from both studies were combined (Fig. 1B; Table 1), median lifespan was again significantly higher (X^2^ = 16.480, P<0.0001) in female *Irs1^−/−^* mice compared to WT controls. Maximum longevity was also increased in this combined data set: the lifespan of the longest lived 10% of mice was significantly greater (X^2^ = 5.629, P<0.05) in *Irs1^−/−^* mice (1187±31 days) relative to WT animals (973±36 days). Median lifespan was also significantly increased (X^2^ = 5.059, P<0.05) in male *Irs1^−/−^* mice relative to WT controls (Fig. 2; Table 2).

**Table 1:** Comparative survival characteristics of female wild type (WT) and *Irs1^−/−^* mice.

	Genotype	Median	Mean	Range	N
**Current study**	WT	789	782±27	572–1019	16
	*Irs1^−/−^*	869	837±64	257–1228	15
**Original study**	WT	738	748±32	343–1049	21
	*Irs1^−/−^*	971	950±38	693–1207	14
**Combined**	WT	760	763±21	343–1049	37
	*Irs1^−/−^*	922	891±39	257–1228	29

Lifespan is reported in days (± s.e.m., where appropriate) for WT and *Irs1^−/−^* mice from this current study and our original study [12]. Combined = Combined lifespan data derived from current study and original study. N = sample size.

**Table 2:** Comparative survival characteristics of male wild type (WT) and *Irs1^−/−^* mice.

Genotype	Median	Mean	Range	N
WT	775	786±21	432–1042	37
*Irs1^−/−^*	896	897±41	619–1192	12

Lifespan is reported in days (± s.e.m., where appropriate) for WT and *Irs1^−/−^* mice. N = sample size.

In conclusion, we replicate and validate our previous finding [12] that global deletion of *Irs1* induces a robust lifespan extension in female mice. Therefore, we now clearly show that, in addition to the GH deficient dwarf mice [17], [18], [19], [20], [21], [22], female *Irs1^−/−^* mice also show a highly consistent and repeatable lifespan extension across distinct studies. In common with the Ames dwarf mouse lifespan studies [17], [18], the two separate lifespan studies in female *Irs1^−/−^* mice were undertaken in the same vivarium, albeit at different times. We suggest that replication of survival data, such as ours and those of the GH dwarfs [17], [18], [19], [20], [21], [22], will significantly help increase confidence in exactly what genetically modified mice are valid models of retarded aging [13], [14]. In turn, this confidence should focus time and resources on those interventions that will ultimately help us to understand what mechanisms underlie healthy aging in humans. It should be noted that one additional limitation in mouse longevity studies is that they are generally performed in a single genetic background, e.g. C57BL/6. We suggest that it will be highly informative for future studies to examine longevity in mice, such as *Irs1* nulls, across different genetic backgrounds.

We also show that, in contrast to our earlier study [12], a completed and augmented lifespan study in male mice indicates that median lifespan is extended in male *Irs1^−/−^* mice by ∼16%. This new finding suggests that the lack of any effect was probably due to an incomplete and slightly underpowered earlier study in males [12], with larger sample sizes undoubtedly helping to detect relatively subtle changes between genotypes [23]. This robust lifespan extension across genders in *Irs1^−/−^* mice is consistent with findings reported for other manipulations including dietary restriction [24], [25], rapamycin treatment [6] and GH/GH-receptor deficiency [8], [23], but is not seen in other sufficiently powered longevity studies (e.g. [26]). We suggest that these new findings indicate that global deletion of IRS1 is a *‘replicable and robust phenomenon’* [*13*] that extends lifespan in both female and male mice. Therefore, we suggest that this model can be used with confidence to explore the mechanisms underlying healthy ageing in mammals.

## Materials and Methods

### Ethics statement

All efforts were made to ameliorate suffering following previously described husbandry protocols (see [12]). The experimental procedures described were carried out following local animal ethical committee review (University College London, London UK.), following guidelines set out by 1986 UK Home Office Animal Procedures Act under the Home Office Licence PPL70/6648.

### Animal husbandry

Mice were maintained under exactly the same experimental conditions and protocols as previously described [12], [16], [26]. In brief, *Irs1* ^−/−^ and wild-type (WT; *Irs1^+/+^*) control littermates were generated from heterozygote parents maintained on a C57BL/6 background following 10 backcrosses. Mice were housed in groups of three to eight same-sex littermates at ∼22°C and on a 12-h light/dark cycle (lights on from 0700–2100hrs) under specific pathogen-free conditions [12], [16], [26]. Mice had *ad libitum* access to chow [2018 Teklad Global (5% fat, 18% protein, 57% carbohydrate, and 20% other components) Rodent Diet; Harlan Teklad, Bicester, Oxfordshire, UK] and water. Kaplan-Meier survival curves were constructed using known birth and death dates (see [12], [16], [26]) and the log-rank test used to evaluate statistical differences between genotypes. Cox regression analysis was employed to determine whether date of birth, parental identity, genotype or experimental cohort (experiment 1 [12] or current experiment) influenced lifespan significantly in female mice. Maximum life span was calculated as mean age of the oldest 10% of mice per genotype. The three *Irs1* ^−/−^ male mice that were alive and censored at 756, 756 and 828 days of age in the original study [12] lived until 995, 933 and 1192 days of age respectively. The 2 WT control males alive and censored at 756 and 993 days of age in the original study [12] lived until 968 and 1042 days of age respectively. The age at death of the two additional *Irs1* ^−/−^ male littermates used in this current study were 763 and 1051 days of age and their WT control littermates were 734 and 736 days of age.
